# Barriers and facilitators to integrating mental health and psychosocial support into economic inclusion programming for displaced families in Ecuador

**DOI:** 10.21203/rs.3.rs-8682674/v1

**Published:** 2026-02-20

**Authors:** Daniela Vergara, Arianna Moyano, Amaleah Mirti, Annie G. Bonz, Adriana Monar, Efrén Estudillo, Sara Vaca, Andrea Armijos, Jeremy C. Kane, Franco Mascayano, Yescárleth Rodríguez, Matthew Schojan, Kathleen J. Sikkema, Ezra Susser, Mike Wessels, M. Claire Greene, Kathryn L. Lovero

**Affiliations:** Columbia University Mailman School of Public Health; Columbia University Mailman School of Public Health; Columbia University Mailman School of Public Health; HIAS; HIAS Ecuador; HIAS Ecuador; HIAS Ecuador; HIAS Ecuador; Columbia University Mailman School of Public Health; New York State Psychiatric Institute; HIAS Ecuador; HIAS; Columbia University Mailman School of Public Health; Columbia University Mailman School of Public Health; Columbia University Mailman School of Public Health; Columbia University Mailman School of Public Health; Columbia University Mailman School of Public Health

**Keywords:** Mental health and psychosocial support, Poverty alleviation, Economic inclusion, Refugees and migrants, Implementation determinants

## Abstract

**Background:**

Displaced populations have increased rates of mental health problems, which have been shown to have a bidirectional relationship with economic insecurity. Despite growing calls globally to integrate mental health and psychosocial support (MHPSS) with economic inclusion (EI) programming, few models exist for practical implementation. We co-developed and piloted the *Building the Future Toolkit* (i.e., the Toolkit), an integrated MHPSS-EI intervention for displaced families in Ecuador, a country that hosts a large number of displaced persons. Here, we present participant and implementer perspectives on the barriers and facilitators to implementation of the Toolkit.

**Methods:**

Fifty displaced families in Quito participated in a pilot trial of the Toolkit between October 2023 and May 2024. Following completion of the trial, we conducted six focus group discussions (n=31; 22 intervention participants, 9 staff) to explore determinants of implementation outcomes, guided by Proctor’s Implementation Outcomes Framework and the Consolidated Framework for Implementation Research 2.0. Data were analyzed thematically using a hybrid inductive-deductive approach.

**Results:**

Both intervention participants and staff viewed the Toolkit as highly acceptable, appropriate, feasible, and usable, with strong potential for sustainability. Key facilitators included the holistic integration of mental health and livelihood skills, enhanced social network building among displaced families, the Toolkit’s standardized yet adaptable structure, and alignment with organizational values and existing activities. Barriers included competing economic demands, childcare responsibilities, transportation costs, limited participation of men and youth, and challenges related to donor reporting indicators. Contextual disruptions—such as Ecuador’s 2024 insecurity and energy crisis—posed additional challenges but also demonstrated the Toolkit’s adaptability in unstable environments.

**Conclusions:**

The *Building the Future Toolkit*illustrates how integrated MHPSS-EI programming can be designed and implemented in humanitarian contexts through participatory methods. Our findings highlight the multilevel determinants supporting successful implementation and promoting sustainability. Future integrated program efforts should address structural participation barriers, develop strategies to target engagement of men and youth, and work with funding institutions to create indicators that recognize the interdependence of mental health and livelihoods.

## Background

As of late 2024, over 120 million people globally have been forcibly displaced owing to conflict, violence, human rights violations, and other humanitarian emergencies (1). Displaced populations frequently endure precarious circumstances, encountering barriers to accessing health services, employment opportunities, and basic necessities (2). These social and structural challenges significantly impact the mental health of displaced people (3, 4), resulting in high rates of mental health problems among displaced populations (5–7).

Economic security and livelihood opportunities are key determinants of mental health among displaced persons (8, 9). The relationship between economic insecurity and mental health problems has been shown to be bidirectional, wherein adverse mental health hinders a person’s livelihood, and economic instability exacerbates psychological distress (10). As such, humanitarian health and protection organizations have increasingly called for integrated economic inclusion (EI) and mental health and psychosocial support (MHPSS) programs for displaced populations (11, 12). Despite evidence supporting their effectiveness (13–22), there is limited data on how to implement integrated programs and thus they have not been routinely deployed in most settings (23, 24).

Ecuador, due to its proximity to some of the largest-scale recent displacements in Latin America from Colombia and Venezuela, has become host to a large population of asylum seekers, refugees, and other migrants in need of international protection (hereafter referred to as displaced persons). As of 2025, Ecuador hosts 26,695 refugees (6%), 13,005 asylum seekers (3%), and 429,469 (92%) people in need of protection (25). A large portion of this population face challenges related to irregular migration status, which creates challenges for socioeconomic integration.

To address the growing need for multisectoral integration, we co-developed and pilot tested a multisectoral strategy for integrating MHPSS into EI programming for displaced persons in Ecuador, called the *Building the Future Toolkit* (i.e., the Toolkit). The Toolkit improved mental health and economic outcomes and was considered by both implementers and participants to be highly acceptable, appropriate, feasible, and usable (26). Here, we present an in-depth, qualitative analysis of the barriers and facilitators to implementation of the Toolkit as well as their relationship with trial outcomes.

## Methods

### Study design

The study was conducted in collaboration with HIAS, a non-governmental organization that provides protection services, including EI and community-based MHPSS to displaced persons and host communities. The HIAS EI program, Socioeconomic Support Program (SESP), is a poverty alleviation intervention derived from the Graduation Model Approach and contextualized for emergencies (27). Following the technical global standards for humanitarian settings (12), HIAS also provides a range of MHPSS services with a multilevel care structure, including individual support, through specialist services; group psychosocial support to strengthen coping/social skills and to promote generation of support networks; community programming to connect people to community and family supports through training promoters and local integration activities; and strengthening the capacities of public officials and humanitarian actors to ensure continuity of care and access to services using rights-based and participatory approaches^1^.

We employed a mixed-methods approach to develop and subsequently assess the integration of HIAS MHPSS and SESP components. The full study methodology is published elsewhere (26). Briefly, we used a participatory, iterative process that included displaced persons, HIAS MHPSS and EI program staff, and other stakeholder groups to conceptualize the strategy to integrate MHPSS and EI programs, design and operationalize the strategy and its components, and refine the strategy to fit the context. Between October 2023 and May 2024, the integrated MHPSS and EI program (i.e., *the Toolkit)* was piloted in two HIAS field offices located in Quito. A group of twelve EI and MHPSS personnel—including social promoters, EI advisors, and MHPSS staff (Table 1)—received training on toolkit delivery. One household focal point was enrolled from each of the 50 families participating in SESP (25 families per field office). Families were selected by HIAS based on Venezuelan or Colombian nationality (regardless of migration status) and a Self-Reliance Index score between 2 and 2.7, a scale measuring self-sufficiency from zero to five. The program prioritized inclusion of families led by young adults aged 17–29, larger households with three or more children, and families with members experiencing social protection vulnerabilities such as histories of gender-based violence, marginalized sexual or gender identities, disabilities, chronic illnesses, or pregnancy/breastfeeding. Data collection and study procedures were carried out by two research assistants trained in psychology and experienced in participatory, community-focused research involving displaced populations. Data across study phases were gathered and analyzed in Spanish. Participants provided informed consent prior to involvement, and the study protocol received ethical approval from the Institutional Review Boards at Columbia University (AAAU8002) and Universidad San Francisco de Quito (2023-011E).

### Qualitative data collection

Six months after program implementation, we conducted focus group discussions with SESP participants, purposively sampled to ensure a diversity of genders and ages as well as participants who had attended toolkit activities with different levels of frequency. We also conducted focus group discussions with HIAS staff from EI and MHPSS programs, including social promoters, psychologists, and EI advisors. We recruited each participant by phone two weeks prior to focus groups. Of the 34 SESP participants and HIAS staff invited to focus groups, n = 2 (5.9%) declined to participate and n = 2 (5.9%) were unable to be contacted.

Focus group guides were designed to identify implementation determinants of study outcomes, whose selection was guided by Proctor’s Implementation Outcomes Framework (28) and included acceptability, adoption, appropriateness, feasibility, reach, retention, and usability. For each outcome, guide questions explored barriers and facilitators across the Consolidated Framework for Implementation Research (CFIR) 2.0 domains (29). Each focus group discussion was facilitated by the two trained, Ecuadorian research assistants, one acting as the facilitator and one as the notetaker, and lasted approximately 90 minutes. All focus groups were digitally audio recorded.

### Qualitative data analysis

Focus group discussion recordings were each transcribed by a member of the research team and then uploaded to Atlas.ti for coding. Data was analyzed using a hybrid thematic analysis approach, combining deductive and inductive methods (30, 31). An codebook was developed *a priori* based on guide questions and study implementation outcomes; emergent codes identified during analysis were organized by study outcome. Two members of the research team individually coded each transcript and met weekly to compare coding and revise the codebook as needed. Each code was then summarized and examined for patterns, triangulating results based on different participant perspectives, which yielded themes related to implementation outcomes. Themes were then organized within the five CFIR domains. Discrepancies were resolved via consensus. Following completion of analysis, data were translated to English by the two bilingual members of the research team who conducted coding (Additional Table 1).

## Results

Of the focus group participants who were program staff (k = 2; n = 9), eight (88.8%) were female and all nine were from Ecuador. The average age was 36 years old. For the SESP participants (k = 4; n = 22), we recruited n = 20 people that attended the toolkit activities, but two of them arrived with their partners. As the study unit was the family, the presence of the partners was an opportunity to gather additional perspectives rather than only from the focal point, and thus they were included. SESP FGD participants were 77.3% (n = 17) female and 14 (63.6%) were born in Venezuela while the others were born in Colombia (n = 8, 36.4%). We identified barriers and facilitators to each implementation across all five CFIR domains: innovation, individuals, inner setting, outer setting, and implementation process (Table 2).

### Innovation Domain

Regarding the Toolkit itself (i.e., the *Innovation*), both SESP implementers and participants felt it was highly acceptable, appropriate, feasible, and usable, with strong potential for future sustainability. They highlighted that the holistic and integrated approach of addressing mental health along with developing economic skills helped participants to learn new abilities related to building their social network, integrating into Ecuadorian culture and life, building economic opportunities, and using these to feel empowered in their current situation and explore future opportunities that may further promote sustainable livelihoods.

I think it helps you to look for other things that you like, such as entrepreneurship, to earn the money that is needed. It doesn’t seem true, but it is. Here, they make us leave our comfort zone and we feel good. For example, they helped us to visualize where we were and where we wanted to be and what I need to do to get where I want to be.

– Female, Venezuelan SESP participant

Staff and SESP participants highlighted the social network building among SESP families as one of the main strengths of the Toolkit.

From what I have been able to observe in the SESP, [comparing] the one I led last year and the one I am leading today, they are very different. Because although it was a group of families, they were treated individually. They may have participated together in some workshops or other activities, but that was as far as our intervention went. Now, however, I see that we have been able to create networks, and they are very natural networks between people as they share more spaces and areas of interest. So it is very nice to see in the workshops how they already know each other, how they share. Something that I always told the group is that I see a lot of connections among them, [participants saying] look, I know how to do this, I can help you with this, go this way. So, they share a lot of information and turn into this support.

– EI Advisor, HIAS

Staff and SESP participants also noted that the Toolkit’s inclusion of activities that SESP participants can replicate independently at home further strengthened self-reliance and social networks, and reduced dependence of SESP participants on HIAS.

But I also believe that beyond that, as the methodology strongly points to the issue of building support networks, it is the people themselves who support each other and they can do so own their own. And in this sense, the institution or the organization or the technician is no longer indispensable for that family process.

– MHPSS staff, HIAS

Social network building not only was between SESP participants, but also between HIAS staff. They mentioned that implementation of the Toolkit enhanced their sense of connection and coordination as a team, strengthening their collaborative efforts and encouraging adoption.

Through the methodology that has now been integrated as part of the SESP, I believe that it has allowed us as a team to connect more and at the same time it is visible as a result in the people who are part of the process. You do not see how I am only addressing the individual area, as my colleague mentioned, but [all MHPSS and EI activities] as something integral, I repeat that word “integral”, and not only as a specific area.

– MHPSS staff, HIAS

One recommendation to increase acceptability and appropriateness of the Toolkit was to include more activities to promote families’ integration into the host community.

Because integration is really outside, it is also important to put our feet on the ground because integration is in the community and we have not seen that.

– MHPSS staff, HIAS

Regarding the delivery of the Toolkit, staff appreciated the option of individual and group activities, mentioning that the groups are more useful when addressing common interests, but the individual approach is also important for when group scheduling is not possible or one family has additional needs.

I really like the part that you can do individually or in groups. So, it is also very important to emphasize that, because it may be that we need to review something again with a family, and there is the toolkit that gives us the opportunity to review it individually.

– EI advisor, HIAS

However, SESP participants suggested that group activities should be held with fewer people to promote interaction among them.

Recommendation, perhaps to make [groups] with fewer participants in order to have more space to share, to get to know each other, to interact with everyone, because sometimes in the meetings there are so many of us and we have to do the activity. Sometimes we lose the chance to get to know each other, to talk, maybe I can help him, maybe she can give me guidance for certain things, so we can have this greater warmth with fewer people.

– Female, Venezuelan SESP participant

Staff felt that the Toolkit’s standardized methodology clarified their roles within the SESP, allowing them to save time on activity planning and more effectively conduct activities with participating families.

[The Toolkit] has actually made it easier, not that it has been an added burden or affected the work we do. Rather, a tool that made it easier to carry out our work. I go back to the issue of having a standard, a guide. So, rather than hindering or giving us more burden, it is facilitating.

- Social promoter, HIAS

The level of detail and clarity of the Toolkit was also highlighted as a strength that allowed it to be delivered by both mental health specialists and non-specialists. SESP participants proposed that, in the future, community members could even facilitate activities with their neighbors.

There were and are, as I was also saying at the time, people who could have that heart that we really like, not only to serve, but also to help people. If you understand me as HIAS does, then maybe it would not be a bad idea if HIAS could train some external people who could give this support to other people [in our community].

– Male, Colombian SESP participant

Similarly, SESP staff shared that the Toolkit would be able to be applied in future SESP cohorts, in other HIAS protection programs, as well as in the community.

We also mentioned that it can be applied with individual attention, group attention, or spaces in the community, and I would even dare to think that maybe in a community leadership promotion program, why not work with leaders so that they can also understand what the tool is about and they can also replicate it.

– MHPSS staff, HIAS

Finally, staff considered the continued use of the Toolkit feasible because the implementation didn’t required many additional material resources, but did highlight that it would need a dedicated budget to cover the limited additional costs.

I believe that this is the interesting thing about the methodology, that it does not require a large investment in materials, because economic resources are required to have an impact. But, for example, it does not require an additional technician, so it is so friendly, so accessible, at least for the team, that the resources would be limited.

– MHPSS staff, HIAS

### Individuals Domain

Roles and characteristics of innovation deliverers and recipients, i.e., HIAS staff and SESP participants, also influenced acceptability, appropriateness, reach, and retention. SESP participants shared that the main reason they missed some toolkit workshops was competing priorities, including taking care of their children and work-related difficulties, such as not being paid for the hours they don’t work and the appearance of last-minute jobs.

In fact, I don’t come to the activities so much because of that, because [my employers] don’t give me permission, I have to ask for permission from one month to the next.

– Female, Venezuelan SESP Participant

Implementation staff confirmed that competing priorities were the main reason for participants to miss Toolkit activities and also noted that sometimes participants didn’t go because they couldn’t afford to pay the bus fare, or they felt tired balancing all their obligations.

What I have seen in difficulties are the families who, because of their work situation and activities, have not given themselves the opportunity to participate. It’s like, if I work every day and I have a [free] half day or a whole day, I want to rest, be at home, be with my family. Themoment that you ask them to participate in activities, the answer usually is that they want to rest.

– Social Promoter, HIAS

However, SESP participants shared that, despite facing daily challenges that could hinder their opportunity to participate in Toolkit activities, they were highly motivated to engage because of the importance of the knowledge gained to their families’ economic and general wellbeing as well as their commitment to completing the program.

*I believe that every workshop provides a learning experience. So, I’m always like “I’m going to go” because when I can’t, it’s because there really is something that doesn’t let me. But, I always go because I always leave with something new to apply for myself or for my family*.

– Female, Colombian SESP participant

They also shared that they had a common need for support networks and the program offered an opportunity for building community and reducing isolation.

I had a classmate, the last workshop we did, and she said to me ‘I like to come here because one of the characteristics that we migrants have is that we are alone here.’ So, we come and we all kind of share and we feel good. She told us that and I also think the same.

– Female, Venezuelan SESP participant

Additionally, participants valued the focus on self-reliance, rather than just monetary assistance, and felt that the program was more acceptable and appropriate because it focused on developing skills to empower them to cope with daily life.

Above all, I believe that the most important thing, beyond the economic help I can receive, is the teaching, because that is something priceless. Beyond money, I believe that the most important thing is knowledge, because if one does not really learn how to do things, then it is complicated to accomplish them.

– Female, Venezuelan SESP participant

However, some participants described men and children having a limited opportunity to engage in Toolkit activities because of their other responsibilities, such as work and school.

There is always going to be a group left behind, in this case it would be the children or partners… normally when they come to meetings they ask if they have to bring the children, even if they make them miss school, but the idea is to not make them miss school. And of the husbands, let’s say that there are 4 or 5 husbands, there are no more, they are all mothers who are heads of household.

– Social Promoter, HIAS

### Inner Setting

Participants reported organizational characteristics related to acceptability, appropriateness, feasibility, reach, and sustainability at the level of the inner setting, i.e., HIAS Ecuador. HIAS staff reported that the Toolkit was an acceptable and appropriate fit with the institutional values and feasibly complemented existing activities by providing additional structure.

I think the activities gave more shape to the SESP program… it gave us more of a foundation, what steps to take here, what can work here. I think that the skills that we have been developing, in my case as facilitator, I have used a much more participatory approach, where it is not so much imposed as this is how the business plan is made and we are going to complete it, but rather it is born from the people through the methodologies themselves. This has been a very interesting process, and I feel that it has provided what has always been desired, a real significant learning. And I know that, if they have to make another business plan, this one will be much more relatable than any other methodology that would have been used.”

– EI Advisor, HIAS

However, staff felt that a potential barrier to sustainability beyond the research period is that the integrated work they complete for the Toolkit is not recognized in the specific indicators of work required by MHPSS or EI sector donors.

*I have found it challenging … to ensure that the interventions that we were going to develop are in line with what we have to do in the different areas. For example, there are EI indicators that we are directly involved in but do not add to the* MHPSS indicators.

– MHPSS staff, HIAS

HIAS staff noted that engagement in the Toolkit activities was facilitated by leveraging the community spaces that HIAS already used for implementation of other activities.

I also believe in the importance of being able to mobilize the team [into communities]. That is, not in the sense of motivating the staff, but in the sense of being able to reach the population. The first workshop was held here in the office, the second workshop was held in a community space.

– Social Promoter, HIAS

Reach was further facilitated by participants’ consideration of HIAS as a safe space with a welcoming, supportive, well-trained staff.

So for me that has been very important since I arrived here, I mean all the people who work here and that I have met who have made these spaces have been very kind to me…. it is like that one fills you, they make you want to come, so all these spaces you want to be here because of the attitude that you have with us.

– Female, Colombian SESP participant

### Outer Setting

In January 2024, a crisis emerged in Ecuador leading to an internal armed conflict declaration and deepening the social, economic, political and energy crisis that the country had been facing in the last couple of years. SESP participants mentioned that the insecurity affected them in different areas of their lives. They described how the situation reminded them of those that led them to leave their country of origin and acknowledged feeling anxious, fear, and stress about their family’s safety.

I really felt like, don’t tell me that once again I have to look for a place to go, because this is not living. I escaped from that in order to not live that again, no.

– Female, Venezuelan SESP participant

Moreover, staff noted that stigma against migrants and refugees rose in response to the crisis.

They began to be labeled. There was a moment when the press started to say, it’s migrants who are causing these situations. They are Venezuelans, they are Colombians. This was not the real situation, but it began to stigmatize the foreign population.

– Social Promoter, HIAS

Along with insecurity, Ecuador started facing power outages of up to 8 hours a day, limiting participants’ ability to be economically productive.

Personally, I was affected by the power cuts, especially when they raised it to 8 hours. It was terrible, because the girl who lives in my house and I were working on an order we had received for several blouses—some tops, some dresses—and because of these cuts, we could not meet the planned timeline for delivery, especially because the cuts are during the day, so we had to look elsewhere for that income that we lost over that.”

– Female Venezuelan SESP participant

Staff mentioned that the situation of insecurity and power outagesmade them focus on emergency interventions rather than the Toolkit activities, and they had to restructure the schedule of the Toolkit activities.

With what happened after the event of the kidnaping on the television channel, many processes fell apart, everything became more acute, there was a lot of re-victimization, a lot of re-experiencing of traumatic experiences.

– MHPSS staff, HIAS

The flexibility of the toolkit, however, allowed participants to continue benefitting from the activities even during unstable times. Many staff turned to remote delivery for participants who feared leaving their home or lacked resources to attend.

More than a challenge, I believe that the opportunity, in my case at least, was to work on the applications of these activities remotely, because activities and everything were already planned, so we did it and it went well. Video calls were made for follow up and activities… so yes, I think this new methodology that could be applied was also an opportunity.

– Social promoter, HIAS

### Implementation Process

Process determinants that facilitated the acceptability, appropriateness, and feasibility of implementation included strong technical support by research assistants, training that progressively developed foundational then technical skills over time, and staff participation in the development of the Toolkit, which allowed staff to improve their understanding of multisectoral concepts outside of their specific area (e.g., MHPSS or EI)and to tailor activities to their SESP participants.

Many concepts that we thought we understood, we really didn’t. The topic of self-efficacy and those things opened for discussion the topic of food security, the topics of economic inclusion. I think that having that discussion allowed us to have a broader vision, [to develop] an intervention that attacks a lot of those components. So, I think it was very educational for the design of the methodologies in general with the whole team.

– MHPSS staff, HIAS

While not involved in development, SESP participants also noted that the Toolkit felt more tailored to respond to their needs than other activities.

I have also realized that you had certain activities planned for us, and from these spaces you sort of extracted or determined what was missing, as if to cover other needs [in this new program].

– Female, Venezuelan SESP participant

## Discussion

The *Building the Future Toolkit*, an integrated MHPSS and EI program for displaced persons, was considered by both staff and participants to be highly acceptable, appropriate, feasible, and usable with strong reach and retention (26). In the present qualitative analysis, we identified the barriers and facilitators across CFIR domains that influenced the Toolkit’s implementation and potential sustainability. Our results illustrate both the practical pathways and the complexity of multisectoral strategies designed to simultaneously strengthen psychosocial wellbeing and economic self-reliance.

SESP staff and participant feedback reinforced the well-documented bidirectional relationship between mental health and economic insecurity among displaced populations (3,10,32,33). A recent review of MHPSS and livelihood interventions in humanitarian settings identified 28 previously delivered programs, though only 25% of them combined MHPSS and livelihood interventions into a single program (34). Our participants highlighted the Toolkit’s integrated, holistic approach as key to its accessibility and impact among SESP participants as well as the feasibility of delivery by HIAS staff. Consistent with prior research, participatory co-design of the integrated approach fostered alignment with organizational mission, program goals, and shared ownership among staff (20,35), while the Toolkit’s standardized yet flexible structure reduced planning burden, clarified roles, and allowed for delivery with fidelity by non-specialists or potentially community members (23,36–38). This combination of standardization and adaptability enhanced feasibility under unstable conditions, as demonstrated during periods of insecurity when remote delivery was necessary. Social impacts were an additional critical factor of the Toolkit’s success. Participants highlighted the Toolkit’s role in strengthening social support systems and described HIAS as a safe and trusted space, particularly for women, which facilitated engagement (39). Staff also noted that overall coordination within implementation teams was improved through Toolkit delivery, further supporting sustainability.

From the participant perspective, competing economic demands, childcare responsibilities, transportation costs, and the daily struggle for income often limited consistent attendance, challenges that are well documented in similar humanitarian contexts (40). Despite these obstacles, participants’ strong motivation to learn skills that enhance self-reliance and reduce isolation indicates that carefully tailoring scheduling, providing childcare, or compensating opportunity costs may serve as important implementation strategies. Staff noted that, although activities aligned with institutional priorities and existing workflows, the lack of formal recognition in donor reporting frameworks, particularly the absence of indicators for integrated and intersectoral activities, posed a barrier to securing long-term resources as well as advocating for broader adoption of intersectoral programs (12). At the systems level, contextual shocks such as political instability, widespread power outages, and rising xenophobia further constrained implementation and threatened participant engagement (41). Together, these findings underscore the structural and contextual barriers that integrated interventions must navigate to achieve sustainability.

Taken together, these results offer transferable lessons for other integrated interventions. Participatory co-design processes are essential for alignment with institutional missions and implementer buy-in (39). Balancing standardized, replicable frameworks with context-driven flexibility allows programs to maintain fidelity while responding to changing circumstances. Designing interventions deliverable by non-specialists or community members strengthens scalability, particularly in resource-constrained humanitarian settings (42). Social support and safe spaces play a critical role in engagement and reducing isolation among marginalized groups. Finally, long-term sustainability depends on donor recognition of integrated outcomes; flexible funding mechanisms and multisectoral measurement tools are needed to incentivize cross-sectoral programming and assess the full benefits of integration, both in terms of psychosocial wellbeing and economic self-reliance (12).

This study has several limitations. Findings are specific to two urban sites in Quito and may not generalize beyond the HIAS Ecuador implementation context or similar humanitarian settings. Additionally, the study period coincided with an acute national crisis, which both tested and limited implementation in ways not necessarily replicable in more stable contexts. Finally, data were selfreported and subject to recall or social desirability bias, and minor nuances may have been lost in translation from Spanish to English. However, the use of bilingual team members and rigorous translation protocols largely mitigates this concern.

## Conclusions

This study is among the first to describe determinants of implementation and sustainability of integrated MHPSS and livelihood programs. The *Building the Future Toolkit* demonstrated clear potential for feasibly addressing the interlinked challenges of economic insecurity and poor mental health among displaced families. However, scale-up and sustainability requires dedicated resources for integrated programming, flexible delivery models, and indicators that capture cross-cutting outcomes. Future research should examine the generalizability of implementation determinants identified here in other populations and programs. Rigorous mixed-methods implementation studies across diverse settings could help refine integration models, explore cost-effectiveness, and build an evidence base to advocate for their sustained adoption by donors and host governments.

## Supplementary Material

Supplementary Files

This is a list of supplementary files associated with this preprint. Click to download.

- ADDITIONALFILES.docx

## Figures and Tables

**Table 1:** Type and role of HIAS staff involved in program delivery and focus group discussions.

Social promoters	Social workers and community development professionals. Involved in monitoring SESP participants’ progress, managing cash assistance, providing guidance in access to rights (i.e., health, education, legal advice), organizing trainings and workshops, and strengthening community partnerships.
MHPSS staff	Psychologists responsible for the multilevel MHPSS interventions. Involved in mainstreaming the mental health and psychosocial support approach into other sectors, in this case SESP.
Economic Inclusion Advisors	Professionals in business administration, economics, human resources, or international relations. Responsible for developing tailored economic inclusion strategies and providing technical guidance in wage and self-employment processes to SESP participants.

**Table 2:** Summary of implementation determinants by CFIR domain and implementation outcome affected.

CFIR Domain	CFIR Construct	Theme	IOF Outcome(s) Impacted
Innovation	Innovation design	Holistic, integrated approach builds skills and resilience for diverse situations	Acceptability, appropriateness
	Innovation design	Builds social network between migrant families	Acceptability, appropriateness
	Innovation adaptability	Inclusion of activities that can be replicated independently by families outside of the SESP program	Acceptability, appropriateness
	Innovation relative advantage	Integrated activities increase staff connection and coordination across mental health and economic inclusion	Acceptability, adoption, appropriateness
	Innovation design	Limited interaction with the host community	Acceptability, appropriateness
	Innovation adaptability	Flexibility to apply in individual or group format, based on user needs	Acceptability, appropriateness, feasibility, usability
	Innovation relative advantage	Provides more detailed guide to achieve program objectives	Acceptability, feasibility, usability
	Innovation design	Clarity of content and flexibility allows delivery through different programs and by people of diverse backgrounds	Feasibility, usability
	Innovation cost	Limited material resources needed for delivery	Feasibility, sustainability
Individuals	Recipient motivation	Importance of knowledge gained to family’s wellbeing	Reach, retention
	Recipient need	Opportunity to build support network among other displaced people	Reach, retention
	Recipient need	Opportunity to develop self-reliance	Acceptability, appropriateness
	Recipient opportunity	Other responsibilities limiting men’s and children’s engagement	Reach
	Recipient opportunity	Competing priorities like work and childcare	Retention
Inner Setting	Mission alignment	Fit with institutional values	Acceptability, appropriateness
	Compatibility	Compliments and gives structure to existing activities	Acceptability, feasibility
	Incentive systems	Toolkit activities not counted in indicators used by traditional funders	Sustainability
	Available resources - Space	Leverages existing spaces used for other HIAS activities	Reach
	Culture - Recipient centeredness	Staff are welcoming, supportive, well-trained	Reach
	Compatibility	SESP program targets primarily women, not men	Reach
Outer Setting	Critical incidents	Internal conflict and economic crisis increase migrant mental health problems and stigma	Reach, Adoption
	Critical incidents	Widespread power outages	Reach, Adoption
Implementation Process	Tailoring strategies	Involvement of staff in Toolkit development	Acceptability, appropriateness, feasibility
	Engaging deliverers	Strong technical support by research team	Adoption, feasibility
	Engaging deliverers	Training that built strong foundational and specific technical skills	Adoption, feasibility

Abbreviations: CFIR = Consolidated Framework for Implementation Research 2.0; IOF = Proctor’s Implementation Outcomes Framework

## Data Availability

Primary data collected as part of this study will be made available upon reasonable request to the corresponding author.

## Abbreviations

CFIR: Consolidated Framework for Implementation Research
EI: Economic inclusion
IOF: Proctor’s Implementation Outcomes Framework
MHPSS: Mental health and psychosocial support
SESP: Socioeconomic support program
